# Chronic cavitary pulmonary aspergillosis in an immunocompetent child

**DOI:** 10.1016/j.mmcr.2022.07.001

**Published:** 2022-07-14

**Authors:** Adeyinka A. Davies, Ireti A. Adegbite, Patricia E. Akintan, Usman O. Ibrahim, Abiola O. Adekoya, Rita O. Oladele

**Affiliations:** a Department of Medical Microbiology & Parasitology, College of Medicine, University of Lagos, Lagos, Nigeria; b Department of Medical Microbiology & Parasitology, Olabisi Onabanjo University Teaching Hospital, Sagamu, Ogun State, Nigeria; c Department of Medical Microbiology & Parasitology, Lagos University Teaching Hospital, Lagos, Nigeria; d Department of Paediatrics, College of Medicine, University of Lagos, Lagos, Nigeria; e Department of Paediatrics, Lagos University Teaching Hospital, Lagos, Nigeria; f Department of Radiology, Olabisi Onabanjo University Teaching Hospital, Sagamu, Ogun State, Nigeria

**Keywords:** Aspergillus IgG, Cavitary lung lesions, Chronic pulmonary aspergillosis, *Mycobacterium tuberculosis*

## Abstract

Chronic pulmonary aspergillosis (CPA) is a progressive and destructive disease of the lung parenchyma. We report a 9-year-old boy diagnosed with CPA with a positive Aspergillus IgG and chest imaging of cavitary lung lesions. He was treated with oral Itraconazole with significant improvement. This shows that an index of suspicion should be heightened in the paediatric population with cavitary lung lesions because not all cavitary lung lesions are caused by *Mycobacterium* tuberculosis.

## Graphical abstract

**Figure:**
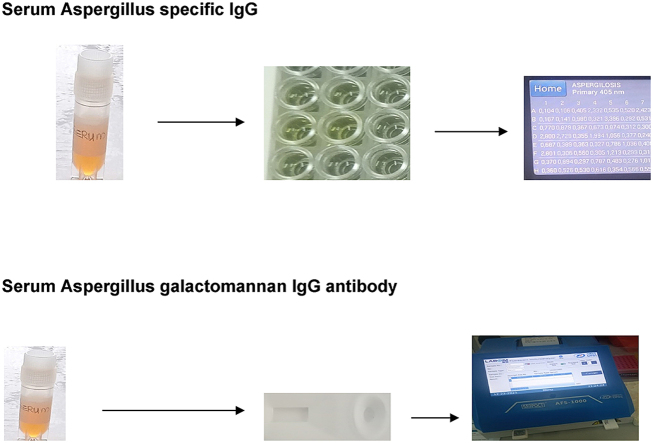

## 1 Introduction

Chronic pulmonary aspergillosis (CPA) is a slowly destructive pulmonary disease characterized by progressive cavitation, fibrosis, and pleural thickening [1]. It occurs in immunocompetent or mildly immunocompromised individuals with underlying respiratory disorders. These may be secondary to *Mycobacterium* tuberculosis, non-tuberculous Mycobacterium (NTM), sarcoidosis, allergic bronchopulmonary aspergillosis (ABPA), and lung surgery [2]. About three million cases of CPA occur annually {3]. It has been listed by the Global Action Fund for Fungi Infections as a priority fungal disease of public health importance [3]. In Nigeria, the estimated prevalence of CPA amongst the smear/gene Xpert negative adult population is 8.7% [4].

The pathogenesis for CPA is poorly understood. However, the failure of conidia clearance, inhalation of a large number of spores and structural defects in the lung parenchyma are probable aetiologies for CPA development [2,5,6]. It may present as aspergilloma, aspergillus nodules, chronic cavitary pulmonary aspergillosis (CCPA), chronic fibrosing pulmonary aspergillosis, and subacute invasive pulmonary aspergillosis [7]. Chronic pulmonary aspergillosis manifests with hemoptysis, cough, and dyspnoea which is similar in presentation to pulmonary tuberculosis [8]. Its diagnosis involves a combination of clinical features, chest imaging findings, and laboratory investigation [9]. The chest imaging findings may include cavitations, pleural thickening, peri-cavitary infiltrations, and/or lung nodules [2]. Rare cases of CPA misdiagnosed as pulmonary tuberculosis and pneumonia have been reported in immunocompetent and immunodeficient children with chronic granulomatous disease and hyper-IgE syndrome [[10], [11], [12]]. Untreated CPA in patients with respiratory disease can be life-threatening, leading to significant deterioration in their quality of life with a case fatality of 75–80% in 5 years [13]. In Africa, the availability and access to essential diagnostics and medications for most fungal diseases remain a challenge [13]. The majority of CPA in Nigeria are in the adult population with sparse data on the paediatric population. We hereby report the diagnosis and treatment of CPA in an immunocompetent child in our hospital.

## 2 Case report

A 9-year-old boy was admitted to our health facility on account of recurrent cough, abdominal pain, and fever of one-week duration (Day 0). The abdominal pain was severe (pain score 9 out of 10), generalized, with associated high-grade intermittent fever, but had no nausea, vomiting or diarrhoea. The cough was non-productive, insidious in onset and non-paroxysmal. It was associated with tachypnoea, chest pain, and drenching night sweats. There was no history of contact with an adult or adolescent with chronic cough. He lived in a self-contained room with his parents, a sibling and a niece in a low-cost area. There was no significant past medical history, except for recurrent cough which was treated with cough syrup and antibiotics.

Physical examination revealed an acutely-ill looking child in obvious respiratory distress with respiratory rates of 28 cycles per minute. He was febrile (temperature 39 °C), dyspnoeic with no peripheral lymphadenopathy but had widespread coarse crepitations in both lung fields. The abdomen was minimally distended with epigastric tenderness. He has hepatomegaly about 6 cm below the right coastal margin. and ascites demonstrated by shifting dullness on percussion. On Day 1, the full blood count showed anaemia (PCV of 21%), leukocytosis with neutrophilia (WBC-18,100/mm^3^, Neutrophil-73%), 18% lymphocytes, 8% eosinophil and mild asymptomatic thrombocytopaenia (platelet- 96,000/mm^3^). Erythrocyte sedimentation rate (ESR) was 58 mm/hr, serum electrolytes showed mild hypernatraemia and alkalosis with moderate hypokalaemia (Sodium 148mmol/l, Potassium 2.97mmol/l, chloride 115mmol/l, bicarbonate 14mmol/l). Viral markers were negative for HIV 1 and 2, and HBV. An abdominopelvic ultrasound scan revealed moderate ascites, hepatomegaly (span 145mm) and normal-sized spleen, adequate for age. Chest radiograph (CXR) showed multiple cavitating nodules in both lung fields (Day 1). A working diagnosis of pulmonary tuberculosis (PTB) was made while we awaited the result of Mantoux and gene Xpert.

**Fig. 1:**
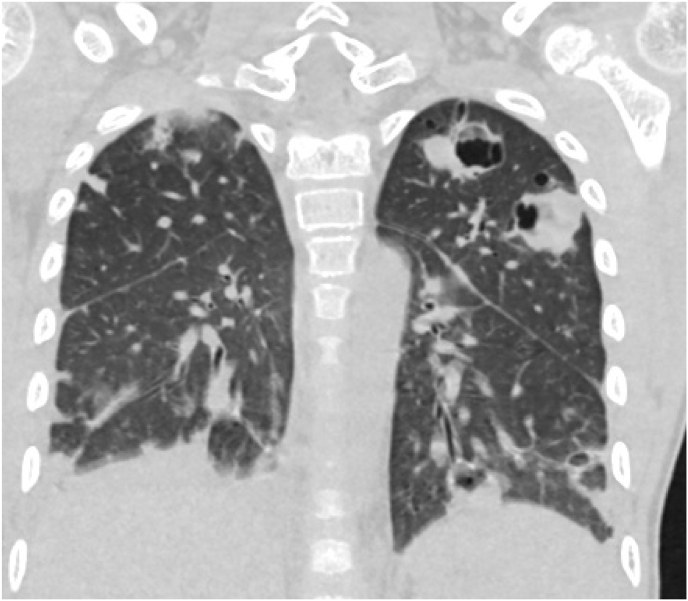
Coronal chest Computed Tomography scan (lung window) showing large irregularly shaped multiple cavities in the left upper lobe with adjacent ground-glass opacity and septal thickening.

**Fig. 2:**
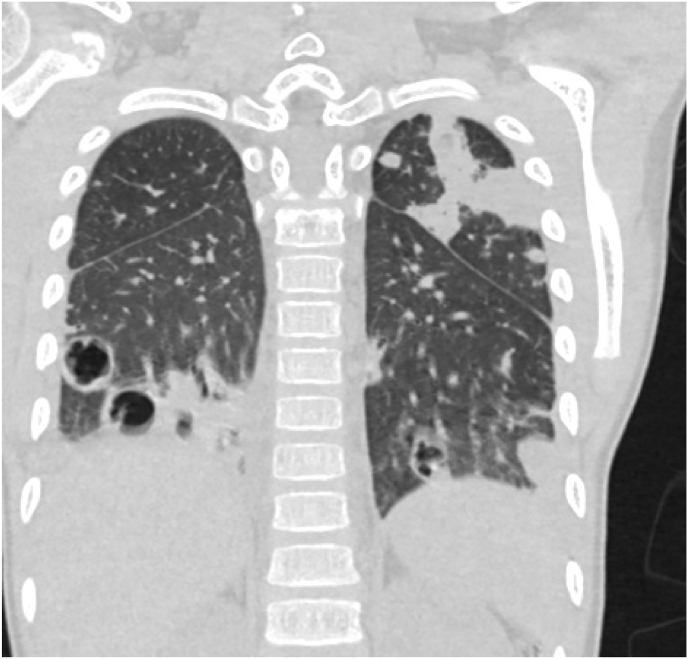
Coronal chest CT (lung window) showing multiple bilateral thick-walled cavitary nodules in both lung lower lobes, septal thickening and thickened pulmonary infiltrates. Left pleural thickening with feeding vessel signs was also noted.

**Fig. 3:**
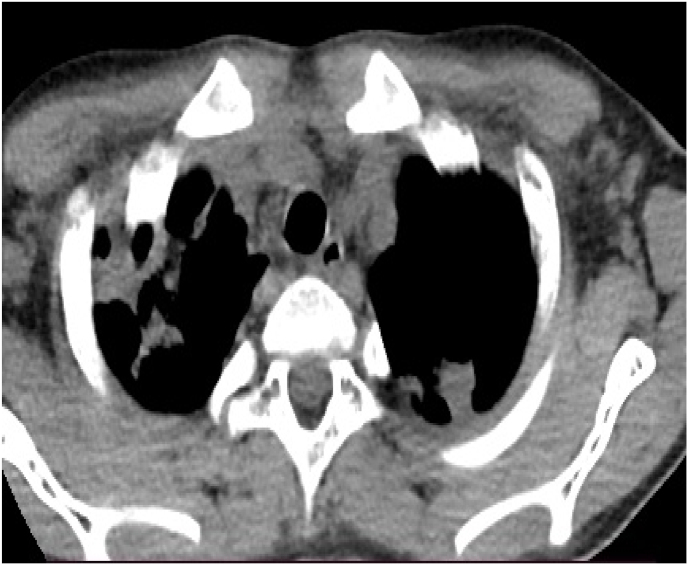
Axial CT of the chest (mediastinal window) showing bilateral pulmonary cavities in the upper lobes surrounded by circumferential pleural thickening.

The patient was transfused with one unit of packed red cell, intravenous antibiotics (ceftriaxone 1g q12h, metronidazole 290 mg q8h (7,5 mg/kg), intravenous fluids and oral potassium were administered for correction of the deranged electrolytes oxygen was administered by nasal prongs/mask at a rate of 4 L per minute on day 2. On day 5, the repeat serum electrolytes were essentially normal (Na^+^ 135mmol/l, K^+^ 3.5mmol/l, Cl^−^ 110mmol/l). Urine microscopy, culture and sensitivity and urinalysis showed no abnormalities. Peripheral blood film showed normocytic normochromic anaemia, with moderate left shift and toxic granulations. Lymphocytes were normal and platelets increased on film and occurred in aggregates. Mantoux test after 72 hours post-injection of purified protein derivative (PPD) yielded no induration (Day 9). Stool for geneXpert was also negative for *Mycobacterium tuberculosis* (Day 10). On day 16, Chest computed tomography (CT) showed multiple thick-walled pulmonary cavitary nodules at the periphery of both lungs with typical feeding signs. Widespread patchy consolidations, ground-glass opacities, and thickened pulmonary infiltrate with pleural thickenings were also noted (Fig. 1, Fig. 2, Fig. 3). These imaging findings necessitated starting the patient empirically on anti-TB medications (Isoniazid/Rifampicin/Ethambutol/Pyranzinamide) (Day 16).

On day 18, the patient developed florid hypopigmented patches on the face and neck with generalized pruritus. Oral fluconazole 150 mg (6 mg/kg OD) and oral loratadine 5 mg daily were immediately added to his medications. Significant clinical improvement was noted after the commencement of these medications; however, with the persistence of fever (Day 21). On day 21, the medical microbiology team was invited. Serum samples for *Aspergillus* IgG antibody, serum galactomannan and urine Histoplasma antigen samples for diagnosis of pulmonary aspergilloma and pulmonary histoplasmosis were taken. *Aspergillus* IgG antibody was increased with a value of 2.795 AU/ML(using Bordier; cut-off of 0.8AU/ML)). Serum galactomannan of 120.93AU/ML (Dynamiker, cut-off of >135AU/ML) and urine Histoplasma antigen of 0.11 ng/ml (IMMY, cut-off >0.20 ng/ml) were negative (Day 22). A definitive diagnosis of chronic pulmonary aspergillosis was made and the patient's previous medications were discontinued and immediately commenced on oral Itraconazole 100 mg twice daily (Day 24). Remarkable improvement in the patient's clinical state was noted within 48 hours of commencing Itraconazole. The fever subsided over 72 hours and the white cell count (WBC- 8000/mm^3^) normalized. On day 28, the patient was discharged home on Itraconazole 100 mg twice daily with regular follow up visits in the paediatrics outpatient clinic.

## 3 Discussion

The criteria for diagnosing CPA require the presence for at least 3 months of the following: the presence of one or more cavities (with or without fungal ball or nodules) on chest imaging, direct evidence of *Aspergillus* infection by microscopy and/or culture of the respiratory specimen with a positive *Aspergillus* specific IgG excluding all other alternative diagnoses [2]. In this reported case, the patient had no prior history of any underlying lung pathology and did not mention prolonged symptoms, however, CXR showed cavitating lung defects, which is highly indicative for a long-standing disorder making the patient at risk for developing lung cavities. Either recurrent bacterial pneumonia, in particular caused by *S. aureus*, or an undiagnosed primary immunodeficiency syndrome might have resulted in the cavitary lung lesions.

Primary immunodeficiency disorders such as chronic granulomatous disorder and hyper IgE syndrome amongst others usually manifest in childhood with recurrent chest infections caused by *S.aureus, Aspergillus species* and other catalase-positive organisms [12,15]. Diagnosis of primary immunodeficiency is based on clinical suspicion together with an immunological work-up including serum immunoglobulin level, T-cell subsets and neutrophil function, amongst others [16]. However, for this patient further investigation for the underlying disorder was not carried out. The preexisting lung cavities most likely has predisposed the patient to CPA. Although bronchoalveolar lavage for fungal culture was not performed, the diagnosis of CPA was made by the radiological abnormalities, the positivity of the Aspergillus IgG assay, and the good response to itraconazole treatment. The severe anaemia was probably due to chronic illness. At presentation, he had hepatomegaly that resolved after transfusion.

Chronic pulmonary aspergillosis commonly occurs after chronic respiratory disorders for which PTB is the most common diagnosis in TB endemic countries [2]. Nigeria is ranked first in Africa and 6th globally amongst the high TB burden countries [17]. Therefore, TB is over-diagnosed and over-treated, especially amongst those with negative geneXpert and suspicious chest imaging as in this index case. Oladele et al. also reported a prevalence of 8.7% amongst smear/GeneXpert negative TB treated patients in Nigeria [4]. This index patient had anti-Koch's therapy commenced despite negative geneXpert result. Chronic pulmonary aspergillosis is most often misdiagnosed as pulmonary TB as reported in several studies [18].In China, cases of CPA misdiagnosed as pneumonia and pulmonary tuberculosis (PTB) amongst four children were reported. Their chest imaging showed lobar consolidation with adjacent pleural thickening and two of which had multiple/solitary small nodules, and another with chronic granulomatous disease [10]. Chronic cavitary pulmonary aspergillosis has also been reported in a 7-month-old girl. The CXR showed bilateral pulmonary inflammation and local consolidation while the chest CT showed a large thick-walled cavity in the right lower lung lobe [19]. This presented case attests that CPA could develop in children and maybe the first reported case in children from Nigeria and possibly Africa.

## 4 Conclusion

Chronic pulmonary aspergillosis is a challenging fungal infection with high morbidity and mortality. It can be found in immunocompetent children and in our locality, hence, not rare. Therefore, there is a need to heighten clinicians’ index of suspicion for CPA in children with radiological findings mimicking pulmonary tuberculosis as not all cavitary lung.lesions are caused by *Mycobacterium* tuberculosis.

## Author contributions

A.P.E and R.O.O took part in the literature review and the critical review of the manuscript. D.A.A was involved in the conceptualization of the case report, reviewed literature and wrote the manuscript. A.I.A reviewed the literature, I. U.O wrote the case report. A.A.O reviewed the chest imaging.

## Sources of funding

There are none.

## Competing interests

There are none.

## Consent

Written informed consent was obtained from the patient or legal guardian(s) for publication of this case report and accompanying images. A copy of the written consent is available for review by the Editor-in-Chief of this journal on request.

## Declaration of competing interest

The authors declare no conflict of interest.
